# An Anti-Cancer Peptide LVTX-8 Inhibits the Proliferation and Migration of Lung Tumor Cells by Regulating Causal Genes’ Expression in p53-Related Pathways

**DOI:** 10.3390/toxins12060367

**Published:** 2020-06-02

**Authors:** Peng Zhang, Yujie Yan, Junting Wang, Xiaoping Dong, Gaihua Zhang, Yong Zeng, Zhonghua Liu

**Affiliations:** The National and Local Joint Engineering Laboratory of Animal Peptide Drug Development, College of Life Sciences, Hunan Normal University, Changsha 410081, China; zhangpeng19911206@163.com (P.Z.); yanyujie@smail.hunnu.edu.cn (Y.Y.); wangjunting1@163.com (Y.W.); dongxiaoping666@163.com (X.D.); ghzhang@hunnu.edu.cn (G.Z.)

**Keywords:** peptide, LVTX-8, cytotoxicity, migration, transcriptomics, nude mouse model

## Abstract

Spider venom has been found to show its anticancer activity in a variety of human malignancies, including lung cancer. In this study, we investigated the anti-cancer peptide toxin LVTX-8, with linear amphipathic alpha-helical conformation, designed and synthesized from the *c*DNA library of spider *Lycosa vittata*. Multiple cellular methods, such as CCK-8 assay, flow cytometry, colony formation assay, Transwell invasion and migration assay, were performed to detect peptide-induced cell growth inhibition and anti-metastasis in lung cancer cells. Our results demonstrated that LVTX-8 displayed strong cytotoxicity and anti-metastasis towards lung cancer in vitro. Furthermore, LVTX-8 could suppress the growth and metastasis of lung cancer cells (A549 and H460) in nude mouse models. Transcriptomics, integrated with multiple bioinformatics analysis, suggested that the molecular basis of the LVTX-8-mediated inhibition of cancer cell growth and metastasis manifested in two aspects: Firstly, it could restrain the activity of cancer cell division and migration through the functional pathways, including “p53 hypoxia pathway” and “integrin signaling”. Secondly, it could regulate the expression level of apoptotic-related proteins, which may account for programmed apoptosis of cancer cells. Taken together, as an anticancer peptide with high efficiency and acceptable specificity, LVTX-8 may become a potential precursor of a therapeutic agent for lung cancer in the future.

## 1. Introduction

Lung cancer is the leading cause of cancer-related mortality worldwide [1]. The prognosis of lung cancer is poor and always misleading, due to the deficiency of symptoms in the early stage [2]. The development of distant metastases accompanied by generalized symptoms in multiple organs is the final stage of lung cancer progression [3]. Traditional treatments, including radiation and common chemotherapy, struggle to effectively and enduringly inhibit the proliferation and metastasis of lung cancer. Thus, there is an urgent need to search for new therapeutic agents for lung cancer therapy. Natural venom, from spiders, insects, snakes and scorpions, is a valuable source for the development of anti-cancer drugs [4,5,6,7]. For example, multiple spider venom components show potential therapeutic activity against cancer, including lung cancer [8,9,10]. Furthermore, naturally occurring anticancer peptides (ACPs), and the corresponding information of sequence structure, provide significant references for the creation of new ACPs in therapeutic applications [11,12]. Multiple bioinformatics tools and combinatorial libraries have been used for the de novo minimalist design of amphipathic peptides, and template modifications can benefit the identification of new ACP sequences [13,14].

*Lycosa vittata* is a medium-sized and venomous spider, mainly distributed in Southwest China [15]. The pharmacological function of its venom remains unexplored so far, especially in the anticancer field. In this study, using the cDNA library of *Lycosa vittata* in our laboratory, we successfully designed and synthesized a linear and amphipathic ACP, entitled LVTX-8. Liner ACPs or anticancer compounds showed powerful anti-metastasis activity against many tumors, both in vitro and in vivo, in previous studies [16]. Liu S et al. found that a specific kind of liner melittin, derived from the bee *Apis mellifera*, could prevent the metastasis of liver cancer cells through inhibition of the Rac1-dependent pathway [17]. Additionally, several previous studies demonstrated that curcumin could inhibit cancer invasion and induce apoptosis in pancreatic cancer and lung cancer, through different molecular mechanisms [18]. Cell-penetrating peptides (CPP) are a class of peptides, with 5–30 amino acids, which could penetrate the cell membrane. They are classified into several types: hydrophobic CPPs, amphipathic CPPs and cationic CPPs. CPPs could across the cell membrane and gain entry into cells through endocytosis and direct translocation. As an α-helix CPP, LVTX-8 could penetrate into cells to promote cell death. The above studies provided significant references for us to investigate the underlying anti-cancer attributes of LVTX-8.

The aim of this study is to investigate the potential abilities of LVTX-8 in inhibiting cell viability and motility in lung cancer, both in vitro and in vivo. The anticancer activities were measured using colony formation assay, CCK-8 assay and flow cytometry, while the anti-metastasis activities were characterized by Transwell invasion assay in A549 and H460 cells. We used subcutaneous and tail vein injection for the preparation of the xenograft and the metastasis model. To examine the variance of causal genes’/proteins’ expression levels and decipher the underlying molecular mechanism of LVTX-8′s anti-cancer attribute, comparative transcriptomics, integrated with multiple bioinformatics analysis, was performed between different experimental models.

## 2. Results

### 2.1. Purification and Characterization of the Peptide LVTX-8

**Figure 1:**
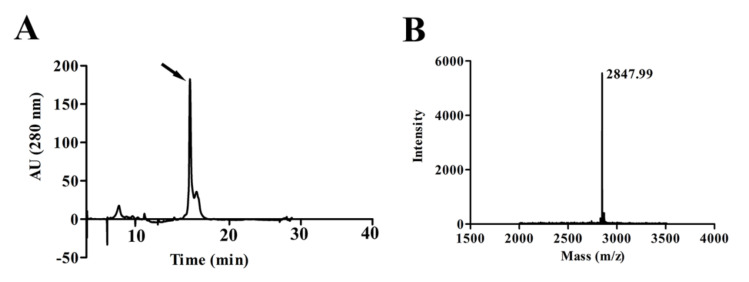
Purification and molecular mass determination of LVTX-8. (**A**) The purification of LVTX-8 using RP-HPLC. (**B**) The molecular weight of LVTX-8 was determined by MALDI-TOF MS.

The *Lycosa vittata’*s cDNA library was constructed in our laboratory. According to the sequence information in the cDNA library and the relevant properties of the anticancer peptides [19,20], we successfully designed and synthesized a linear and amphipathic alpha-helical peptide, which was entitled LVTX-8 (Figure S1A,B). The amino acid sequence of the LVTX-8 is IWLTALKFLGKNLGKHLAKQQLSKL. Using solid-phase peptide synthesis, we successfully prepared the sample of LVTX-8. The synthetic LVTX-8 was purified using C18 Reversed-phase high performance liquid chromatography (RP-HPLC) (Figure 1A). Then, the peak was obtained and analyzed using Matrix-Assisted Laser Desorption/ Ionization Time of Flight Mass Spectrometry (MALDI-TOF MS). The peak for LVTX-8 displayed a molecular mass of 2847.99 Da, which is equal to the calculated value according to the sequences. (Figure 1B). The result indicated that the designed LVTX-8 was successfully synthesized.

### 2.2. LVTX-8 Inhibited Cancer Cell Viability and Colony Formation

**Figure 2:**
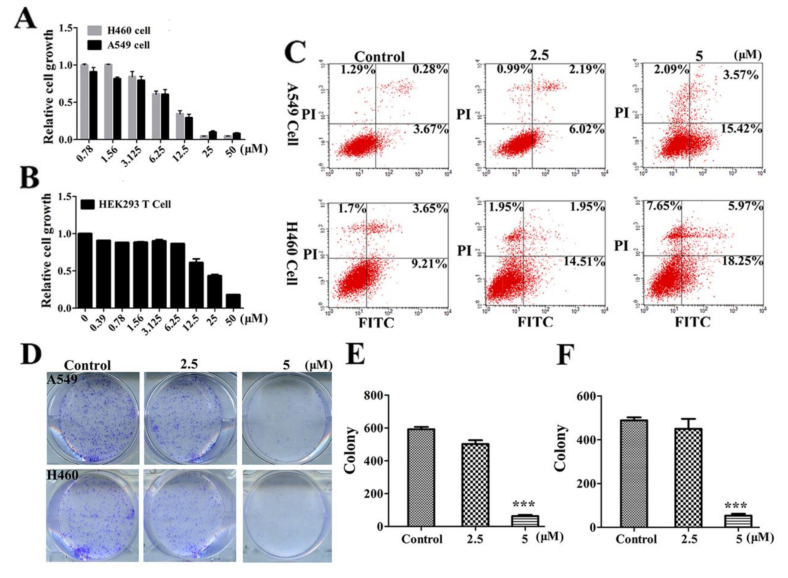
The effect of LVTX-8 on cell cytotoxicity and growth. Effect of LVTX-8 on A549, H460 (**A**) and HEK293T cells (**B**). The cytotoxic activities were detected by CCK-8 assay after treatment with LVTX-8 for 24 h (**C**). A549 and H460 cells apoptosis treatment with LVTX-8 was achieved by flow cytometry. Colony formation of A549 and H460 cells treated with LVTX-8 was evaluated (**D**). The situation of A549 and H460 colony formation with 2.5 μM or 5 μM LVTX-8 treatment. Quantitative results of A549 (**E**) and H460 (**F**) colony are illustrated (*n* = 3).

In order to determine whether LVTX-8 treatment suppresses cancer cell growth, CCK-8 assay was carried out to test the viability of lung cancer cells, including A549 cells and H460 cells. They were treated with various concentrations of LVTX-8 for 24 h. Our results suggested that LVTX-8 exhibited significant cytotoxicity, in both A549 and H460 cells. Then, the 50% inhibitive concentration (IC_50_) of LVTX-8 was examined. The IC_50_ value of A549 and H460 cells was approximately 8 μM after 24 h incubation (Figure 2A). Furthermore, the cell viability of the non-cancer cells HEK293T was examined. As shown in Figure 2B, the IC_50_ value of the non-cancer cells was about 19 μM. These results indicated that LVTX-8 showed strong cytotoxicity, rather than selectivity, in cancer cells. Next, we used an Annexin V-FITC/PI apoptosis detection kit to test the cell viability of cancer cells. The A549 and H460 cells were treated with 2.5 and 5 μM of LVTX-8 for 24 h, respectively. As shown in the flow cytometer data, by comparison with the control cells, the percentage of apoptotic cells increased from 5.24% to 21.08%, and from 14.56% to 31.87%, in 5 μM of LVTX-8-treated A549 cells and H460 cells, respectively (Figure 2C). Subsequently, we selected the concentrations of 2.5 and 5 μM of LVTX-8 for comparative treatment in A549 and H460 cells in the following experiments.

Using the colony formation assay, we further verified the effects of LVTX-8 on cell growth. Compared with the control, LVTX-8 treatment caused a strong inhibition of colony formation in both lung cancer cells in a dose-dependent manner (Figure 2D–F). These results, in conjunction with the CCK-8 assay presented in Figure 2A, suggested that LVTX-8 exhibited high cytotoxicity in both lung cancer cells at low concentrations.

### 2.3. LVTX-8 Suppressed Cell Migration and Invasion

**Figure 3:**
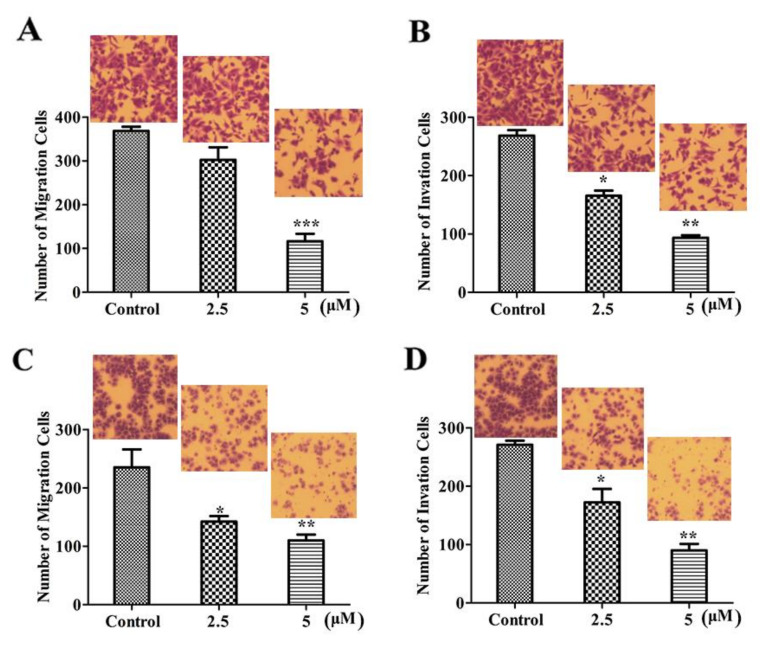
LVTX-8 inhibited A549 and H460 cells’ migration and invasion, detected by Transwell chamber assay. Representative images and statistical analysis of migrated A549 cells (**A**) and H460 cells (**C**) in the Transwell migration assay (*n* = 3). Representative images and statistical analysis of invaded A549 cells (**B**) and H460 cells (**D**) in the Transwell invasion assay (*n* = 3).

Liner ACPs have been reported to have an inhibitory effect on many cancer cells [21,22]. However, their roles in anti-metastasis are not entirely clear. In order to explore the effect of LVTX-8 on cell migration and invasion, Transwell migration and invasion assay were performed. These results showed that LVTX-8 could significantly inhibit cell migration activity in a dose-dependent manner (Figure 3A,C). Similarly, LVTX-8 could impair cell invasion ability in both cell models in a dose-dependent manner (Figure 3B,D). These results clearly demonstrated that LVTX-8 not only inhibited cancer cell growth, but also possessed an anti-metastasis function in lung cancer cells at a concentration below the IC_50_.

### 2.4. LVTX-8 Inhibited Tumor Growth in Tumor Xenografts

**Figure 4:**
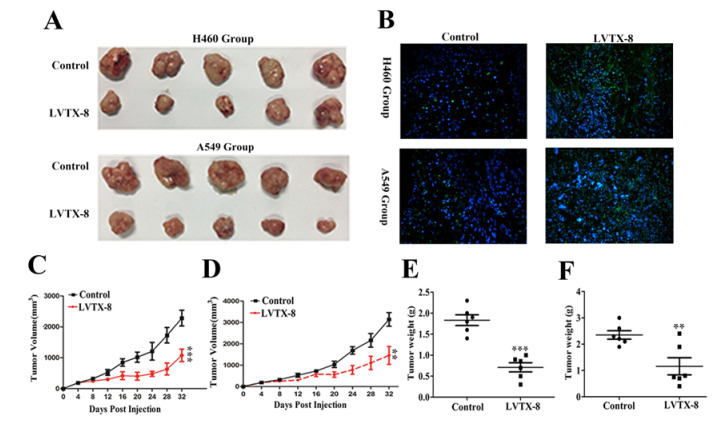
The influence of LVTX-8 on the growth of A549 and H460 in xenograft tumors. (**A**) Images of the nude mice and their xenograft tumors at 32 d after injection (*n* = 5). (**B**) In situ labeling (TUNEL) examination of nude mice tumor tissues. Dynamic volume of xenograft tumors at different times after injection, for A549 xenograft model (**C**) and H460 xenograft model (**D**). Weight of xenograft tumors at the 32nd day after injection, A549 (**E**) and H460 (**F**).

LVTX-8 exhibited considerable anticancer effects on both A549 and H460 cells in vitro. To further determine whether LVTX-8 has the same inhibitory effect in vivo, we performed a nude mice xenograft tumor model experiment. In the view of the fact that LVTX-8 may be easily degraded by multiple proteases in vivo [23], D-LVTX-8 was synthesized. The cytotoxicity of D-LVTX-8 against A549 and H460 cells was similar to that of LVTX-8 (data not shown). Therefore, the LVTX-8 sequences used in the following animal experiments are all D-type amino acid substitutions. PBS(phosphate buffer saline, as a control) injection resulted in an increase of tumor size (Figure 4A), while 10 mg/kg LVTX-8 treatment could significantly suppress the tumor growth and reduce the tumor volume in H460 and A549 groups (Figure 4C,D). Compared with the PBS group, tumor weights in the LVTX-8 group were significantly lower than those in the control group (Figure 4E,F). To evaluate apoptosis in tumors, tumor tissues were analyzed by Terminal Deoxynucleotidyl Transferase (TdT)-mediated dUTP Nick-End Labeling (TUNEL) assays. The blue dots represent nuclei and the green dots represent the TUNEL signals of the apoptotic cells. As shown in Figure 4B, compared to the control subjects, many more green dots (apoptotic cells) were observed for LVTX-8-treated groups in both A549 cells and H460 cells. Quantitative analysis of TUNEL staining of the positive cells in Figure S2 showed that there was a significant difference between the LVTX-8 treatment group and the control group. Taken together, the results suggested a significant inhibitory role of LVTX-8 in lung cancer growth in vivo, through the activation of apoptosis.

### 2.5. LVTX-8 Prevented the Metastasis of A549 and H460 Cells in Nude Mice

**Figure 5:**
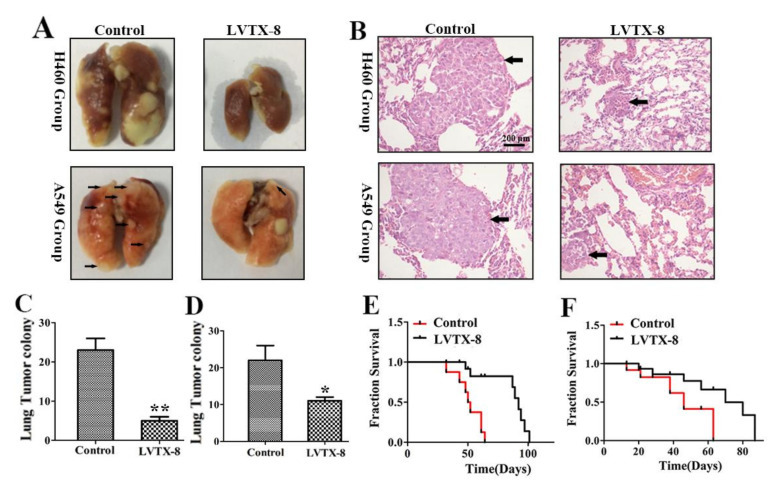
LVTX-8 inhibited metastatic lesion formation. (**A**) Representative lung imaging of nude mice injected with H460 (upper) and A549 (lower) cells. (**B**) H&E examination of nude mice lung tissues that received different treatments. Phosphate buffer saline (PBS) (left), LVTX-8 (right). (**C**,**D**) Quantification of H460 (left grouping) and A549 (right grouping) lung metastatic colony formation of the lung metastasis in the nude mice model (*n* = 5). (**E**,**F**) The Kaplan–Meier method is used to assess the survival time of animals. The survival times of H460 (left grouping) and A549 (right grouping) mice in the LVTX-8-treated group were significantly longer than those in control group (*n* = 6).

The migration and invasion of lung cancer cells was immensely suppressed by LVTX-8 treatment in vitro, and so we wondered if LVTX-8 would have the same effect on tumor growth in vivo. Thus, we performed a lung metastasis experiment via tail intravenous injection [24,25]. According to the types of the injected cells, the metastasis models could be divided into the A549 group and the H460 group. The details of the lung metastasis test and the overall survival test are presented in Table 1. As shown in Figure 5A, the ex vivo image of the lungs indicated that the pulmonary nodules of peptide treatment were fewer than those of the control group. Note that the metastatic lesion does not puncture the lung, but does so massively in the H460 group. There were significant differences between the metastatic lung lesions of the LVTX-8-treated group and the control group. Mice treated with 10 mg/kg LVTX-8 had lower metastatic lesion formation in the lungs (*p* < 0.01) than the vehicle control mice in the H460 group, and a similar effect was found in the A549 group (*p* < 0.05) (Figure 5C,D). In addition, the hematoxylin-eosin staining (H&E) results for the mouse lung tissue in Figure 5B showed that dense cells composing clumps were more obviously found in the control groups as compared to the LVTX-8-treated groups. Furthermore, mice treated with LVTX-8 showed delayed cancer cells metastasis and inhibited tumor growth, which may result in obviously longer survival (approximately extended by 30 days in the H460 group and by 20 days in the A549 group) compared to the control (Figure 5E,F). These data showed that LVTX-8 could significantly decrease the homing ability of A549 and H460 cells in vivo. The above results suggested that LVTX-8 may be a potential drug for targeting lung cancer progression.

### 2.6. Transcriptomics Analysis between Case-Control Samples

**Figure 6:**
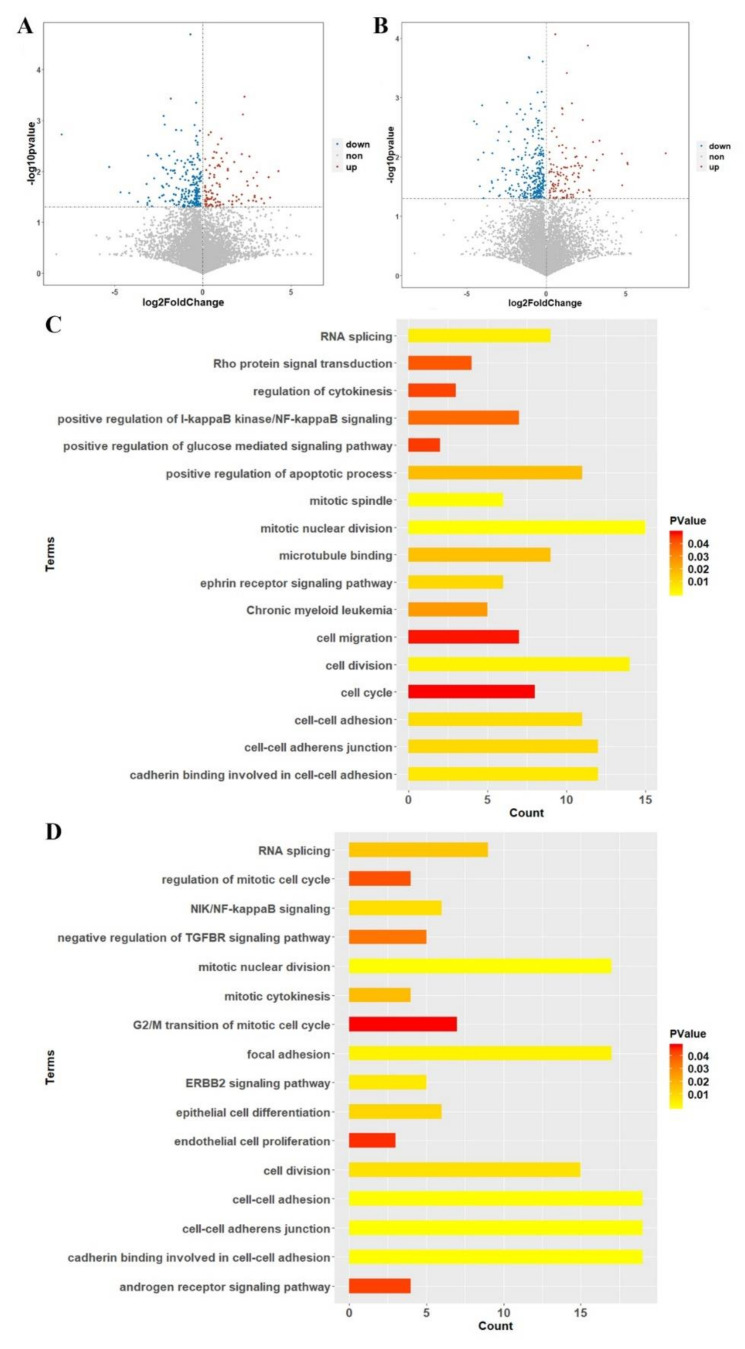
Enrichment analysis based on differentially expressed genes (DEGs) identified in 2 µM and 5 µM LVTX-8-treated samples. (**A**,**C**) show the enrichment analysis results based on DEGs between 2 µM LVTX-8-treated and control samples, while (**B**,**D**) show the enrichment analysis results based on DEGs between 5 µM LVTX-8-treated and control sample.

We compared the Fragments per Kilobase Million (FPKM) of each transcript from the control, 2 µM-treated and 5 µM-treated subjects, respectively. A student’s t-test was used for filtering the differently expressed transcripts and a *p* value of < 0.05 was set as the statistical cut-off line. There were 310 differentially expressed transcripts between the 2 µM LVTX-8-treated and control samples (Figure 6A), and 401 between 5 µM LVTX-8-treated and control samples (Figure 6B).

### 2.7. Gene Enrichment and Pathway Analysis

**Figure 7:**
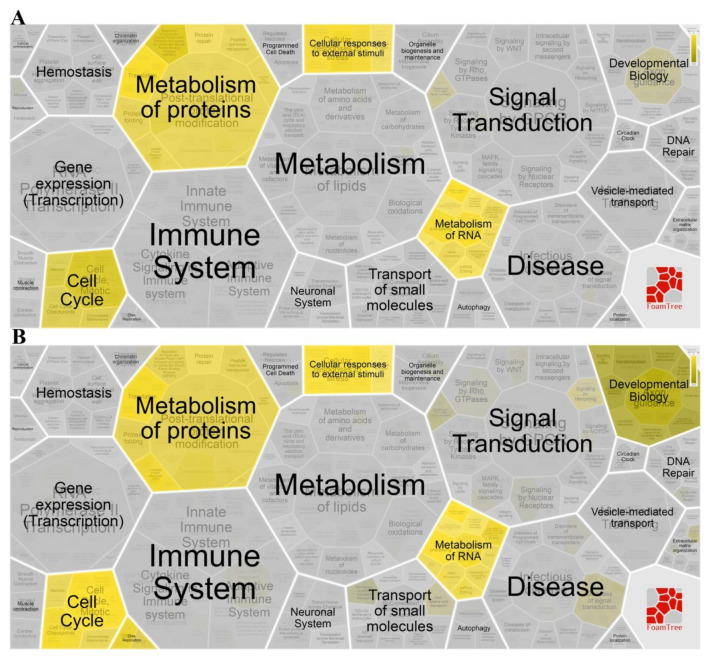
Result of Reactome pathway analysis. (**A**,**B**) showed the main biological processes and hierarchical structure of Reactome pathways (*p* < 0.05) after 2 µM and 5 µM LVTX-8 treatment, respectively.

According to similarity and hierarchy, Reactome pathways were classified into different groups, including cell cycle, metabolism of proteins, programmed cell death, signal transduction, etc. A total of 310 differentially expressed genes (DEGs), between the 2 µM LVTX-8-treated and control samples, were imported into the Database for Annotation, Visualization and Integrated Discovery (DAVID) and Reactome online tools, for gene enrichment analysis and pathway analysis. A total of 75 functional terms and six pathways were generated with *p* < 0.05. As expected, most of the DEGs were enriched in migration, adhesion, cell cycle, mitotic and apoptotic terms (Figure 6C); results of the Reactome pathway analysis showed that LVTX-8 could significantly alter the cell cycle, signal transduction and cellular responses to external stimulation (Figure 7A). Meanwhile, 94 terms and seven pathways were generated, based on 401 DEGs between the 5µM LVTX-8-treated and control samples. Most DEGs were significantly enriched in adhesion, cell cycle, mitotic and signal transduction-related terms (Figure 6D). Reactome pathways were significantly associated with cell cycle, programmed cell death, immune system, metabolism of RNA and vesicle-mediated transport (Figure 7B). The results of Gene Ontology (GO) and pathway analysis demonstrated that LVTX-8 may inhibit cancer cell growth by regulating signal transduction, programmed cell death and cell cycle (Figure 6C–D), and suppress the metastasis and invasion of lung cancer cells by regulating cell adhesion and migration (Figure 6C,D). Our enrichment analysis demonstrated that LVTX-8 could induce cell apoptosis. The results are consistent with those from flow cytometry (in vitro) and the TUNEL experiment (in vivo).

### 2.8. Gene Set Enrichment Analysis (GSEA)

**Figure 8:**
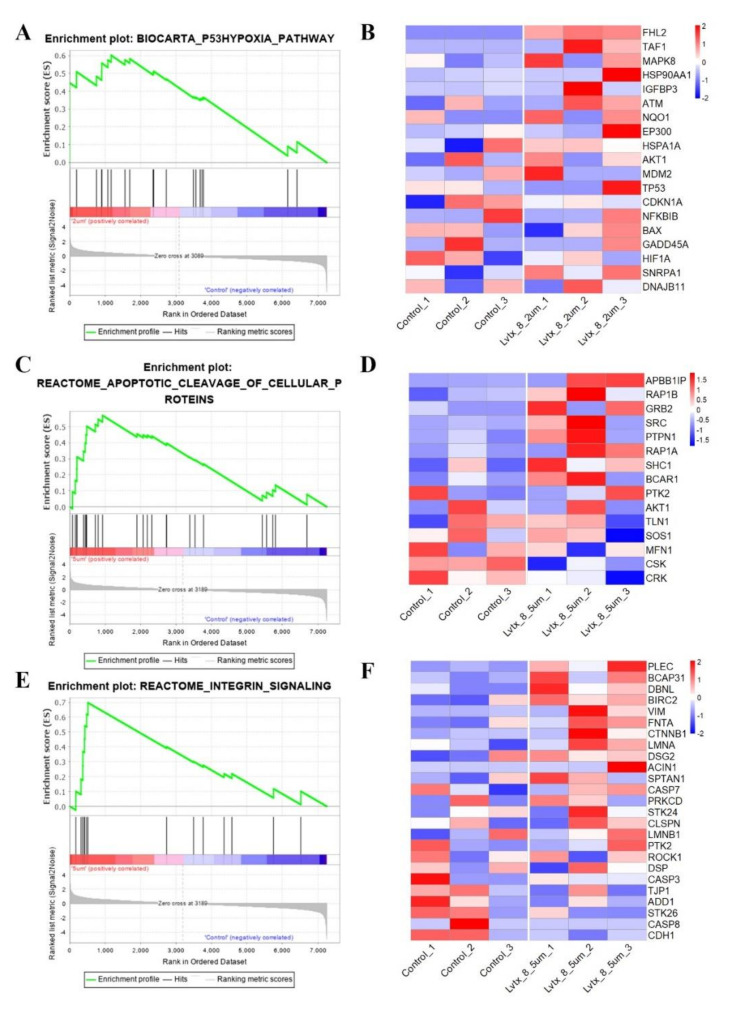
Result of gene set enrichment analysis (GSEA). (**A**) Enrichment plot of P53 hypoxia between 2 µM LVTX-8-treated and control samples. (**B**) Heatmap of get sets in P53 hypoxia pathway based on transcripts expression. (**C**) Enrichment plot of apoptotic cleavage of cellular proteins between 5 µM LVTX-8-treated and control samples. (**D**) Heatmap of get sets in apoptotic cleavage of cellular proteins based on transcript expression. (**E**) Enrichment plot of integrin signaling between 5 µM LVTX-8-treated and control samples. (**F**) Heatmap of get sets in integrin signaling based on transcript expression.

In gene set enrichment analysis (GSEA), the p53 hypoxia pathway was up-regulated in the 2 µM LVTX-8-treated samples (*p* < 0.01, *q* < 0.05) (Figure 8A) and the 5 µM LVTX-8-treated samples (*p* < 0.05, *q* < 0.15) (Figure 8C,E). In this pathway, core genes, including *FHL2*, *TAF1*, *MAPK8*, *ATM* and *NQO1* (Figure 8B), and *APBB1IP*, *RAP1B*, *GRB2*, *SRC*, *PTPN1*, *RAP1A* and *SHC1* (Figure 8D), were significantly up-regulated after 2 µM and 5 µM LVTX-8 treatment. While in another pathway, “apoptotic cleavage of cellular proteins”, core genes *PLEC*, *BCAP31*, *DRNL*, *BIRC2*, *FNTA*, *LMNA* and *DSG2* were up-regulated after 5 µM LVTX-8 treatment (Figure 8F). These results suggested that LVTX-8 could induce p53-dependent apoptosis and cell cycle arrest, and thus suppress A549 cell growth both in vitro and in vivo.

## 3. Discussion

Spider venom is a valuable resource with abundant molecular diversity. Some peptides derived from spider venom are potential candidates for anti-cancer therapy, including lung cancer. There are many similarities between LVTX-8 and two other AMPs: LyeTx-1 and LycoToxin-1 [26,27]. Our study suggested that the *Lycosa vittata*-derived α-helix ACP LVTX-8 has two functions: on the one hand, it inhibits the growth of cancer cells by suppressing division and migration. On the other hand, α-helix ACPs were considered in inducing cell apoptosis against cancer cell [28].

CCK-8 and colony formation experiments showed that LVTX-8 suppresses the survival of the A549 cell in a dose-dependent manner. The amount of apoptotic A549 cells was increased with the increased concentration of LVTX-8. As a typical α-helix ACP, LVTX-8 could target intracellular proteins, like most CPPs, to facilitate A549 cell death [28,29]. Some pathways involved in cell cycles and apoptosis, such as the “p53 pathway”, could be triggered by CPPs [30]. P53 is the most important tumor suppressor, and it can regulate many biological processes, including cell cycles and apoptosis [31]. Enrichment analysis indicated that several processes, including cell cycles, cell division, migration and adhesion, were significantly changed by LVTX-8 treatment (Figure 6C,D). The Reactome pathway analysis showed that the biological processes regulated by the P53 pathway, including cell cycles, gene expression (transcription) and signal transduction, were significantly changed after LVTX-8 treatment (Figure 7). Furthermore, the results from GSEA also indicated that the core genes in the P53 hypoxia pathway were significantly up-regulated (Figure 8A–D). These results could reasonably explain the phenotypes of cancer cells, via CCK-8 assays and colony formation experiments in vitro. To sum up, RNA-seq analysis demonstrated that the growth and migration of cancer cells were inhibited through regulation of cell cycles and apoptosis via the p53 pathway.

In addition, LVTX-8 also showed the same effects on cell growth and apoptosis in vivo. Xenograft tumors were inhibited, and the apoptotic cells were increased, as detected by TUNEL, after LVTX-8-treated. A significant inhibition of tumor metastasis was observed in A549 xenograft nude mice treated with LVTX-8. Metastatic lesion formation in lungs was suppressed after LVTX-8 treatment of nude mice. The result of RNA-seq analysis revealed that migration and adhesion were regulated after LVTX-8 treatment (Figure 8C,D). Causal DEGs were significantly enriched in functional terms, including “cell migration”, “cell–cell adhesion”, “cell–cell adherents’ junction” and “cadherin binding involved in cell–cell adhesion”, both in low and relatively high concentrations for LVTX-8-treated samples (Figure 6C,D). Cadherin is a well-known suppressor of tumor growth and invasion [32]. The results of enrichment analysis demonstrated that cadherin may play a central role in regulating cell apoptosis and adhesion (Figure 6C,D). Impaired cadherin-mediated cell–cell adhesion can promote cell death via triggering apoptosis. Furthermore, CASP7 was also up-regulated in the “apoptotic cleavage of cellular proteins” pathway. The activation of CASP7 played a central role in apoptosis (Figure 8C,D). In addition, our result suggested that cell adhesion and migration were regulated by the “integrin signaling” pathway. Integrin could mediate signal transduction and cell adhesion, and thus regulate cell apoptosis and cell growth. Taken together, sufficient evidence, from the physiological, cellular and molecular levels, supports claims that the anti-cancer peptide LVTX-8 could inhibit cell apoptosis and migration, by regulating the expression of p53 and cadherin-related genes.

In spite of LVTX-8 showing strong cytotoxicity and anti-metastasis activity towards lung cancer cells, including A549 and H460 cells with IC50 values of 8 μM and 19 μM, it also showed this activity towards to non-cancer cells, such as Hek293T cells. This specificity is barely satisfactory. High efficiency and specificity are two important standards for evaluating the potential use of a precursor in drugs. Tumor microenvironment features, including PH value, tissue construction, function and metabolism, could provide significant references for targeted molecular modifications of potential precursors of therapeutic agents. Molecular modification of a peptide with a known sequence includes adding or deleting specific amino acid, and adding functional chemical groups. Further studies will be performed that focus on two points: verification studies of p53 expression levels in LVTX-8-treated cancer cells, and targeted molecular modifications of LVTX-8.

## 4. Materials and Methods

### 4.1. Materials

All Fmoc amino acids and rink amide-aminomethyl (AM) resin was purchased from GL Biochem (Shanghai, China). CCK-8, crystal violet and 4’,6-diamidino-2-phenylindole (DAPI) were obtained from Sigma. The Transwell insert (24-well insert; pore size 8 μM,) and Matrigel were purchased from Corning (New York, NY, USA) and BD Biosciences (San Jose, CA, USA), respectively.

### 4.2. Cell Culture

Human lung carcinoma cells (A549 and H460) were purchased from ATCC (Manassas, VA, USA). The H460 and A549 cells were cultured in DMEM medium and F12K medium (Invitrogen), respectively. All culture medium contained 1% PS (penicillin and streptomycin), 1% Gln and 10% FBS (fetal bovine serum) at 37 °C with 5% CO_2_. PS, Gln and FBS were obtained from Invitrogen.

### 4.3. Peptide Synthesis

According to the literature [33], LVTX-8 (Ac-IWLTALKFLGKNLGKHLAKQQLSKL-amide) was synthesized using the Fmoc SPPS (9-fluorenyl-methoxycarbonyl solid-phase peptide synthesis) method.

### 4.4. Peptide Purification and Mass Determination

The synthetic LVTX-8 was purified using RP-HPLC (C18, 4.6 × 250 mm and 1 mL min^−1^) via the 0%–80% acetonitrile gradient for 48 min. The eluted peptide fractions were obtained and used in Matrix-Assisted Laser Desorption/ Ionization Time of Flight Mass Spectrometry (MALDI-TOF MS) for mass determination. For mass determination, a 1 μL aliquot of eluted peptide fractions and an equal volume of a matrix solution were spotted onto a 96-well target plate. The matrix solution contained 20 mg mL^−1^ α-cyano-4-hydroxycinnamic acid (CCA), 50% acetonitrile (ACN) and 0.1% trifluoroacetic acid (TFA). The monoisotopic molecular mass of each peptide was determined at an acceleration voltage of 25 kV.

### 4.5. CCK-8 Assays

The cells (A549 and H460) were seeded at the density of 1 × 10^5^ cells mL^−1^ in DMEM or F12K culture medium. Aliquots (90 μL) of cell suspension and 10 μL of LVTX-8 at various concentrations were added into a 96-well microplate. After incubation at 37 °C for 24 h, 10 μL CCK-8 was added in the corresponding well and the 96-well microplate was incubated for 4 h. The cell viability was determined using a CCK-8 assay.

### 4.6. Flow Cytometry Analysis

For cell apoptosis assay, A549 or H460 cells were co-incubated with 2.5 μM and 5 μM LVTX-8 for 12 h, respectively. According to the manufacture’s instruction, cells were washed twice with PBS, digested with trpysin, stained with Annexin V-EGFP/PI kit (Keygene Biotech, Nanjing, China) and then analyzed by flow cytometry.

### 4.7. Colony Formation Assay

For examining the survival of A549 and H460 cells treated with LVTX-8, cells were plated (2 × 10^4^ per well) in a 6-well plate and incubated 24 h. After 12 h, cells were digested with trpysin, counted, and seeded into 60 mm dishes in a density of 500 cells per plate. After 36 h, cells were treated with 2.5 μM or 5 μM LVTX-8 under conditions of 37 °C and 5% CO_2_. The peptides were refilled every 7 days for a total 21 days. All the colonies were washed with PBS and then stained with 2% crystal violet.

### 4.8. Migration and Invasion Assay

The migration and invasion abilities of the cells were determined via the Transwell migration and invasion assays, respectively. For the Transwell migration assay, A549 cells or H460 cells were treated with different concentrations of LVTX-8 in 200 μL of serum-free medium in each upper chamber. Volumes of 500 μL complete medium containing different concentrations of LVTX-8 were added into the bottom chamber. After 24 h, the cells in the upper chamber were removed, and the migration cells in the membrane were stained with 2% crystal violet. Under a light microscope, the stained cells were photographed and counted in at least six randomly-selected fields. The method for the Transwell invasion was assay mainly the same as that for the Transwell migration assay, besides the use of a matrigel-coated Transwell insert. All experiments were repeated 3 times.

### 4.9. Animal Model

All the animal studies were approved by the Institutional Animal Care and Use Committee (IACUC) of Hunan Normal University (Number: 006/2019), and the National Institutes of Health guidelines for the performance of animal experiments were followed. There was no obvious influence of sex on the results of the study. BALB/c male nude mice (7–8 weeks old) were purchased from SLACCAS Jingda (Changsha, China). A549 or H460 cells (1 × 10^7^) in Hank’s solution /200 μL were injected into the right flank of each nude mouse by subcutaneous injection, and were grown as subcutaneous tumors. After 15 days, 10 mice divided randomly into each group (A549, H460) were used in these experiments. A quantity of either 0 or 10 mg/kg LVTX-8 was intraperitoneally administrated every other day. After 32 days of treatment, all mice were sacrificed. Transplanted tumors were excised, paraffin-embedded, sectioned and stained for TUNEL to reveal histological architecture.

For preparation of the metastasis model, nude rats were injected with 1 × 10^6^ A549 cells or H460 cells in 200 μL Hank’s solution via the lateral tail vein. Immediately after tumor cell injection, rats were randomly allocated to each group (A549, H460). The drug treatment group was injected i.p. with 10 mg/kg LVTX-8, every other day for 30 days. The control group was injected with PBS, every other day for 30 days. 

### 4.10. RNA-seq Analysis

Total RNA was extracted by use of a mirVana miRNA isolation Kit (ambion) and an Agilent 2100 Bioanalyzer (Agilent Technologies, Santa Clara, CA, USA) for evaluation of the RNA integrity. According to the manufacturer’s instructions, TruSeq Stranded mRNA LTSample Prep Kit (Illumina, San Diego, CA, USA) was used to construct libraries. Then the Illumina sequencing platform HiSeq X Ten (Illumina, Shanghai OE Biotech Co., Ltd.) was used to sequence these libraries. An output of 150 bp paired-end reads was generated. Sequencing of raw data was processed by Trimmomatic [34]. The cleaned reads were mapped to the hg38 reference genome using Hisat2 [35]. The reads were assembled using StringTie. FPKM value of each transcript was calculated using Ballgown. Differentially expressed transcripts were identified using t-test (*p* < 0.05).

### 4.11. Gene Functional Annotation, Enrichment and Pathway Analysis

DAVID is a knowledgebase that includes many tools, which can perform functional annotations and enrichment analyses using large gene lists [36,37]. In order to decipher the underlying molecular mechanisms, all DEGs were imported into DAVID for functional annotation, gene enrichment and pathway analysis.

Reactome is a database that can perform pathway analysis, visualization and interpretation [38]. To obtain the significant biological information, all DEGs were imported into Reactome for pathway analysis. *p* < 0.05 was set as the statistical cut-off line for identification of significant GO terms and pathways.

### 4.12. Gene set Enrichment Analysis (GSEA)

GSEA software (version 4.0.3) was used to perform gene set enrichment analysis [39,40]. Gene sets with *p* < 0.05 and *q* < 0.25 were considered as significant functional modules that could be worthy for further validation studies.

### 4.13. Statistical Analysis.

An independent student’s *t*-test was used for two-group comparisons. Statistical analysis was performed by GraphPad Prism 5.0. * *p* < 0.05, ** *p* < 0.01 and *** *p* < 0.001 were used to show statistical significance.

## Figures and Tables

**Table 1:** Experimental design of metastasis and overall survival test.

Experiments	Lung Metastasis Test				The Overall Survival Test			
Tumor Types	A549		H460		A549		H460	
Treatments	LVTX-8	PBS	LVTX-8	PBS	LVTX-8	PBS	LVTX-8	PBS
Animals	5	5	5	5	6	6	6	6

## Supplementary Materials

The following are available online at https://www.mdpi.com/2072-6651/12/6/367/s1, Figure S1: Use i-Tasser software to predict the secondary structure of LVTX-8., Figure S2: Quantitative analysis of TUNEL staining positive cells in tumor xenografts from mice in various groups.
