# Supplementary material for: An Anti-Cancer Peptide LVTX-8 Inhibits the Proliferation and Migration of Lung Tumor Cells by Regulating Causal Genes’ Expression in p53-Related Pathways
Source: Toxins (Basel). 2020 Jun 2;12(6):367. doi: 10.3390/toxins12060367 (PMC7354478; doi:10.3390/toxins12060367)
Supplement: Supplementary file 1 [file toxins-12-00367-s001.pdf]

An anti-cancer peptide LVTX-8 inhibits the proliferation and migration of lung tumor cells by regulating causal genes expression in specific pathways

Peng Zhang, Yujie Yan, Junting Wang, Xiaoping Dong, Gaihua Zhang, Yong Zeng * and Zhonghua Liu *

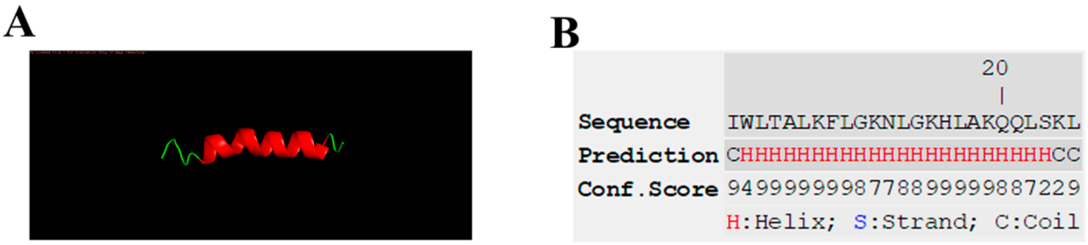

Figure S1. Use i-Tasser software to predict the secondary structure of LVTX-8. (**A**) Diagram of the secondary structure model of LVTX-8. (**B**) Table of prediction results of secondary structure of LVTX-8 from i-Tasser. The red H indicates that the secondary structure is a helix. The blue S indicates that the secondary structure is a strand. The black H indicates that the secondary structure is a coil. Conf. Score indicates the credibility of the prediction.

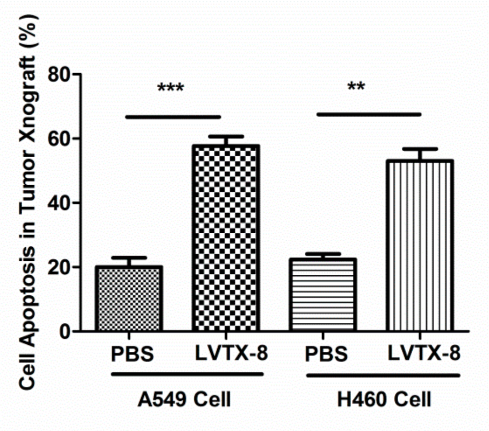

Figure S2. Quantitative analysis of TUNEL staining positive cells in tumor xenografts from mice in various groups.
